# Computational Modeling of Chromatin Fiber to Characterize Its Organization Using Angle-Resolved Scattering of Circularly Polarized Light

**DOI:** 10.3390/polym13193422

**Published:** 2021-10-05

**Authors:** Muhammad Waseem Ashraf, Aymeric Le Gratiet, Alberto Diaspro

**Affiliations:** 1 Nanoscopy and NIC@IIT, CHT Erzelli, Istituto Italiano di Tecnologia, Via Enrico Melen 83, 16152 Genoa, Italy; aymeric.legratiet@iit.it; 2 DIFILAB, Department of Physics, University of Genoa, Via Dodecaneso 33, 16146 Genoa, Italy; 3 Institut FOTON-UMR 6082, Université de Rennes, CNRS, F-22305 Rennes, France

**Keywords:** chromatin fiber, nucleosomes, light scattering, circularly polarized light, discrete dipole approximation

## Abstract

Understanding the structural organization of chromatin is essential to comprehend the gene functions. The chromatin organization changes in the cell cycle, and it conforms to various compaction levels. We investigated a chromatin solenoid model with nucleosomes shaped as cylindrical units arranged in a helical array. The solenoid with spherical-shaped nucleosomes was also modeled. The changes in chiral structural parameters of solenoid induced different compaction levels of chromatin fiber. We calculated the angle-resolved scattering of circularly polarized light to probe the changes in the organization of chromatin fiber in response to the changes in its chiral parameters. The electromagnetic scattering calculations were performed using discrete dipole approximation (DDA). In the chromatin structure, nucleosomes have internal interactions that affect chromatin compaction. The merit of performing computations with DDA is that it takes into account the internal interactions. We demonstrated sensitivity of the scattering signal’s angular behavior to the changes in these chiral parameters: pitch, radius, the handedness of solenoid, number of solenoid turns, the orientation of solenoid, the orientation of nucleosomes, number of nucleosomes, and shape of nucleosomes. These scattering calculations can potentially benefit applying a label-free polarized-light-based approach to characterize chromatin DNA and chiral polymers at the nanoscale level.

## 1. Introduction

Chromatin is a complex of DNA and proteins that exist in the nucleus of eukaryotic cells [1,2]. It plays a primary role in DNA packaging and gene regulation in the cell cycle [3]. During this cycle, chromatin organization changes and has different compaction levels depending on the cycle stage [4]. Knowing this structural organization, we can understand DNA replication and other nuclear processes [5]. The optical microscopy approaches [6,7,8] and cryo-electron microscopy [9,10] have mainly been used to discern the chromatin organization. The optical microscopy methods usually require chromatin-specific fluorescent probes for labeling, and these labels can be invasive [11,12]. Here we adopted one label-free microscopy approach based on the angle-resolved scattering of circularly polarized light to characterize the structural organization of chromatin fiber. Measuring the scattered light from an object, we can infer the size, shape, birefringence, dichroism, and depolarization properties of the object [13,14,15,16]. The 4×4 Mueller matrix contains all the properties of the sample under examination [17,18,19,20]. It is a mathematical tool that relates the incident and the scattered light through the relation: $S_{\mathrm{out}}=\left[M\right]S_{\mathrm{in}}$, where $S_{\mathrm{out}}$ is the scattered light Stokes vector, $S_{\mathrm{in}}$ is the incident light Stokes vector, and M is the Mueller matrix [20]:

(1) $$\left[M\right]=\left[\begin{array}{cccc} m_{11} & m_{12} & m_{13} & m_{14}\\ m_{21} & m_{22} & m_{23} & m_{24}\\ m_{31} & m_{32} & m_{33} & m_{34}\\ m_{41} & m_{42} & m_{43} & m_{44} \end{array}\right]$$

Each element of a Mueller matrix describes some polarization property of the sample [20,21]. However, the interpretation of polarization properties from the individual elements is challenging, and many decomposition methods have been reported to extract information from Mueller matrix [22,23]. Different polarization properties; dichroism, retardance, depolarization, diattenuation have been interpreted from it and employed to characterize various samples in biophysics, nanochemistry, and materials [24,25,26,27,28]. In this study, we focused on the $m_{14}$ element of the Mueller matrix. This element is the circular dichroism (CD), in the absorption band of the sample, that is the differential extinction of right and left circularly polarized light [29]. However, in the scattering regime, $m_{14}$ was reported as the circular intensity differential scattering (CIDS) of light [29,30]; it describes the differential scattering of the incident left- and the incident right-circularly polarized light at a scattering angle θ. Mathematically, it is the normalized ratio of scattered intensities of the incident left- and right-circularly polarized light [29]:

(2) $$\mathrm{CIDS}(\theta )=\frac{I_{L}\left(\theta \right)-I_{R}\left(\theta \right)}{I_{L}\left(\theta \right)+I_{R}\left(\theta \right)}$$

The CIDS angular signature were reported, theoretically and experimentally, to give structural information of various samples [31,32,33,34,35]. In numerically reported results, mainly Born approximation and coupled dipole method were used to compute CIDS [36,37]. In the first Born approximation, the appearance of the CIDS signal for chiral objects depends on the anisotropic polarizability tensor of the scatterer. This approximation does not consider the internal interactions in the scatterer and incorporates anisotropy geometrically by replacing isotropic scatters such as spheres with ellipsoids. For the chromatin structure, we cannot ignore the internal interactions between nucleosomes as these interactions play an essential role in the chromatin compaction [38]. Experimentally, the CIDS has been measured usually by modifying commercial CD spectropolarimeters and also by designing home-build setups [31,33,39]. The CIDS signals usually have weak magnitude in the range $10^{-2}$–$10^{-5}$ [31,40]; however, in principle, CIDS value about $10^{-5}$ can be measured [40]. The studies were reported to enhance the magnitude of CD signal [41,42], and this may potentially be extended in the scattering regime to CIDS signals. The CD was utilized to characterize the various polymers [43,44]. The CIDS was combined with other approaches to give better interpretation of polarimetric data [45,46]. In [46], an experimental approach was reported coupling the CIDS and the expansion microscopy to demonstrate its sensitivity to the organization of biopolymers; this study showed that by improving the distance between chiral groups, the new imaging contrast gives access to a better resolution of the chromatin-DNA organization in situ. The study [39] also reported the CIDS imaging capabilities for the nuclear organization of chromatin DNA inside isolated cell nuclei; they showed the CIDS emission was able to distinguish the difference of compaction inside the nucleus induced by the chirality of the molecules; however, in comparison, the fluorescence emission was more of isotropic. To investigate how the changes in chiral parameters of chromatin fiber induce changes in the CIDS signal, we computationally modeled chromatin fiber to know its organization in the cell cycle.

We comprehensively examined CIDS sensitivity to the variations in various degrees of freedom of a chromatin solenoid numerically using discrete dipole approximation. The effect of variation in the pitch, radius, the orientation of solenoid, the handedness of solenoid, orientation of nucleosomes, number of helical turns, and the extinction efficiency as a function of incident light wavelength was demonstrated. The CIDS response was sensitive to the variations in these parameters related to the compaction levels of chromatin solenoid. The scattering calculations can potentially benefit various chiral analytical and experimental imaging methods in biophysics and chemistry. The simulations method, the chromatin fiber model, and simulations results are presented in the following sections.

## 2. Materials and Method

### 2.1. Computational Method

The discrete dipole approximation (DDA) [47,48,49] method, the ADDA code [50], have been employed to compute angle-resolved scattering quantities. The DDA is a numerically exact method [51] and has successfully been employed to calculate various scattering quantities of different objects from micro- to nanoscale in biophysics [52,53,54] and plasmonics [55,56,57]. This method discretizes the scattering object into an array of polarizable point dipoles and considers the interaction of point dipoles. The minimum number of dipoles required to approximate an object depends on the wavelength of the incident electromagnetic wave relative to the size of the object, the refractive index, and the fine description of the shape of the object. The dipoles acquire dipole moment in response to the incident electric field, also interacting with other dipoles, and in the ADDA code, the polarizability of point dipoles has been calculated using lattice dispersion relation (LDR) [47,50]. The objective of DDA is to calculate the dipole moment of each dipole and other quantities are calculated from the dipole moments. We discretized the chromatin solenoid into dipoles by taking 60 dipoles in the longest dimension of the helical fiber for its one turn; the number of dipoles in the other directions is set by the discretization tool automatically based on the given value [50,58]. All the inner and outer integrations were converged to the stopping criterion value in the ADDA. The default values as given in ADDA v.1.3b4 have been used for all the settings to compute scattering quantities; the stopping criterion, the computation of Mueller matrix, the iterative solver, and the orientation averaging [50]. All electromagnetic simulations were run on the Intel(R) Dell core-i5-8250U CPU @1.6 GHz x64-based processor and 8GB RAM. The scattering problem investigated here is in the usual application domain of DDA where it performs accurately [50], the refractive index condition |m−1|<2 is satisfied.

### 2.2. Chromatin Fiber Model

The actual chromatin organization is still an open question; different models have been proposed with different shapes of nucleosomes [59,60,61]. The emphasis is on the solenoid model of chromatin with nucleosomes shaped as cylindrical units [59,60], and we have adopted this model; other reported nucleosomes shapes are oblate spheroids, spheres, and tetranucleosomal units [61,62].

**Figure 1:**
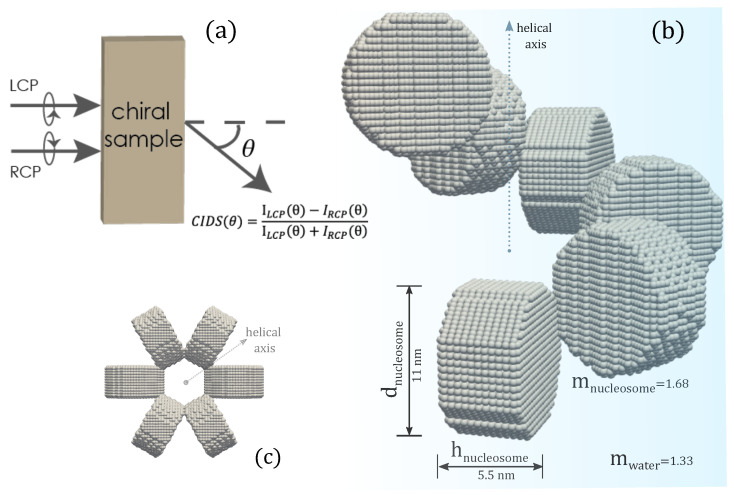
(**a**) Representation of CIDS, (**b**) the discrete dipole model of chromatin fiber, (**c**) projected view along the helical axis.

The chromatin structure we investigated is the solenoid model, a one-start helix, with nucleosomes arranged as a helical array as presented in Figure 1. The 30 nm chromatin fiber composed of cylindrical shaped nucleosomes arranged as a helical array with 6-nucleosomes per single turn has been considered in all the computations. The cylindrical nucleosomes have a height of 5.5 nm, a diameter of 11 nm, and are composed of isotropic dielectric material hosted in water. The refractive index of nucleosomes is 1.68, and that of water is 1.33 [63]. The orientation of nucleosomes is such that their planar faces are parallel to the tangent vector to the helical trajectory and are normal to the helix axis. The discrete dipole model of the solenoid is shown in the inset of Figure 1b.

## 3. Results and Discussions

**Figure 2:**
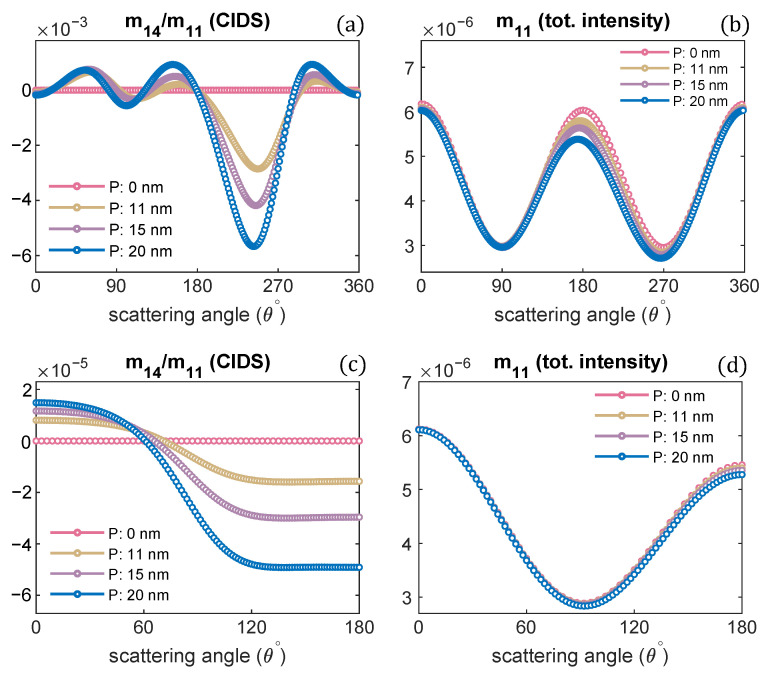
Pitch of chromatin solenoid is varied; the angular behaviour of CIDS and total scattered intensity is presented, respectively: (**a**,**b**) at a fixed orientation (α,β,γ) = (0,0,0), (**c**,**d**) orientation averaging is performed.

The CIDS calculations are performed as a function of scattering angle as its angular dependence gives structural information of chiral objects. The angle-resolved scattering calculations in a fixed orientation are presented in Figure 2a,b.

The orientation defined by Euler angles (α,β,γ) = (0,0,0) is when the incident electromagnetic plane wave propagates along the chromatin helical axis. The pitch of the chromatin solenoid is varied, and the diameter is kept constant. The wavelength of the incident electromagnetic plane wave is 300 nm for these scattering calculations. It is evident from these calculations that the CIDS signal senses the changes in the pitch of the solenoid. This differential scattering signal is identically zero when the pitch of the solenoid is zero, and the magnitude of this signal increases as the pitch of the chromatin solenoid increases. The zero CIDS signal indicates the helical fiber has lost chirality and conforms to a circular array of nucleosomes. The total scattered intensity, however, is not zero for the solenoid when the pitch was zero because it corresponds to the overall size of the solenoid as stated by the calculations in Figure 2b. The total scattered intensity is related to an unpolarized light and the calculations suggest the total intensity is least sensitive to the changes in pitch as compared to the differential scattering signal of circularly polarized light (Figure 2).

In a liquid medium, the helical structures are usually randomly oriented. We have performed orientation averaging to compute the average response over different possible orientations of chromatin solenoid in the water. The structural parameters are kept the same as we simulated for the scattering calculations at a fixed orientation. The numerical results are given in Figure 2c,d. We observe that the CIDS signal is not lost after performing orientation averaging, which suggests a chiral sample in the liquid medium. The existence of CIDS signal in the orientation averaging indicates its capability to characterize the chiral biological structures. In ADDA, if a particle is not symmetric and orientation averaging is not performed, the angle range is extended to $360^{{}^{\circ}}$ for the scattering calculations at a fixed orientation (Figure 2a,b); and for the calculations in the orientation average the angle range to $180^{{}^{\circ}}$ is used (Figure 2c,d).

**Figure 3:**
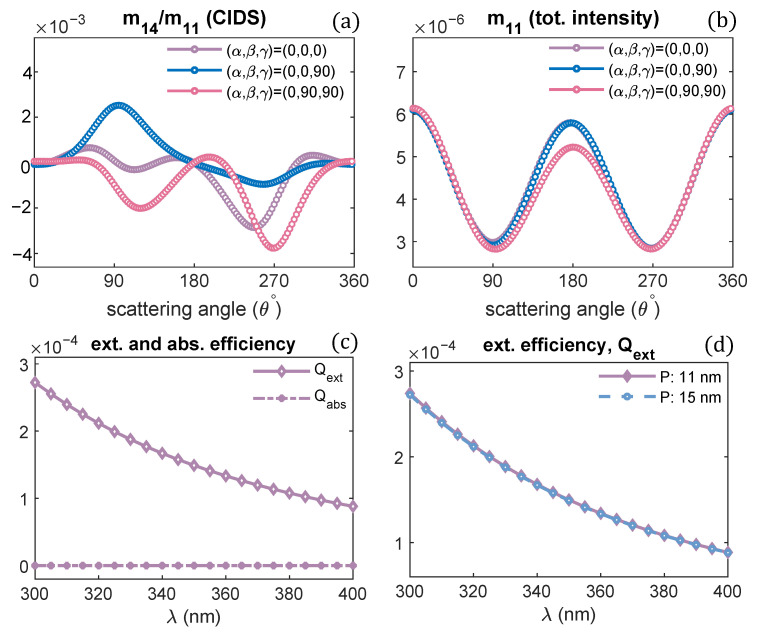
(**a**,**b**) CIDS and total intensity angular dependence for different orientations of solenoid, λ = 300 nm, (**c**) extinction and absorption efficiency of chromatin fiber with pitch 11 nm, (**d**) extinction efficiency of solenoid for different pitch values.

The angular behavior of scattering signal in response to the changes in the orientation of a chromatin solenoid with respect to the incident light is demonstrated in Figure 3a,b.

The structural parameters of a solenoid are kept the same as used for calculations in Figure 2; only the orientation of the solenoid is changed. From the numerical results, we can infer the CIDS signal has a different response for every different orientation of solenoid and depends on the solenoid’s symmetry. In contrast, the total scattered intensity has identically the same behavior for these different orientations because it is related to the size variations and is least sensitive to the symmetry of solenoid. The difference in CIDS behavior in response to changes in the orientation of solenoid is an important indicator that we can distinguish the orientations of chromatin fiber bringing new contrast in 2D imaging. The calculations for the extinction efficiency of the chromatin solenoid are given in Figure 3c,d. This quantity shows the capability of chromatin to remove energy from the incident electromagnetic wave. The extinction efficiency of chromatin decreases at higher wavelengths (Figure 3c); these calculations also show there is no absorption at the simulated wavelength values, and the extinction is due to scattering of light. The calculations in Figure 3d show that the extinction efficiency of a solenoid is least affected by changing its pitch only.

**Figure 4:**
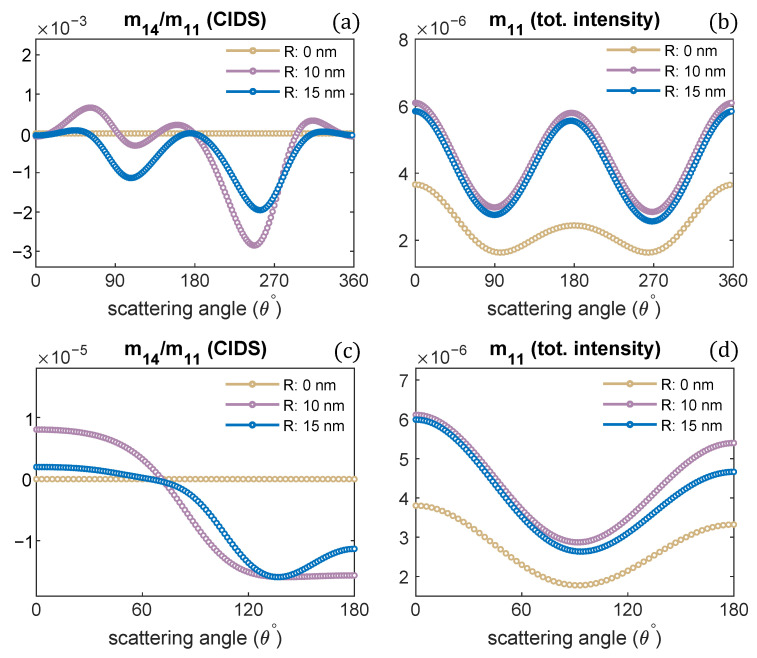
Radius of chromatin fiber is varied, the pitch is kept constant. CIDS and total scattered intensity calculations are presented, respectively; (**a**,**b**) at fixed orientation, (**c**,**d**) orientation averaging is perfromed. P = 11 nm, λ = 300 nm.

Next, we investigated the effect of changes in the radius of the chromatin solenoid on the angular behavior of scattering quantities as presented in Figure 4. The CIDS signal is identically zero for a linear chain of nucleosomes; that is, when the radius is zero and nucleosomes are stacked up along the helix axis. In this case, the solenoid loses its chirality, and this conformation represents the beads on a string chromatin model, the least compact form. The CIDS signal is most significant when the radius is 10 nm, then increasing radius while keeping the pitch of solenoid constant, the CIDS decreases in magnitude as the solenoid is becoming more of circular nature (Figure 4a,c).

**Figure 5:**
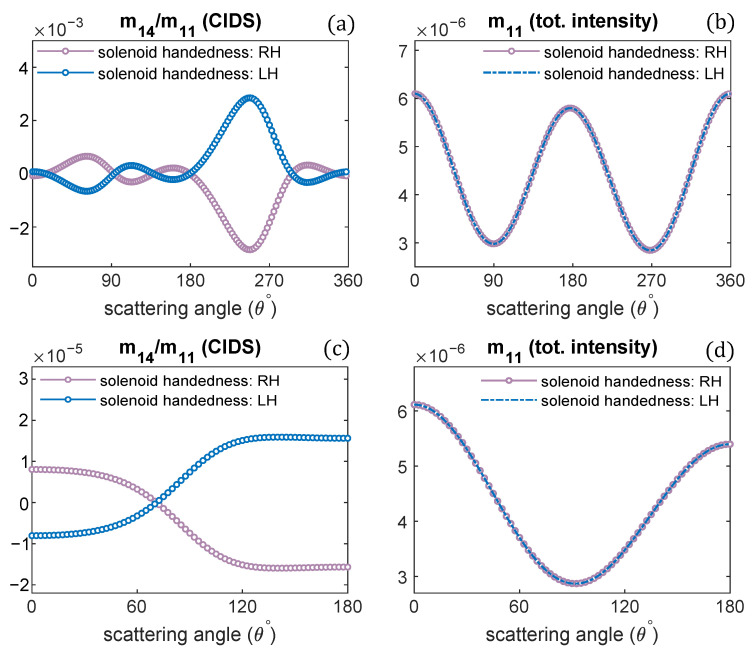
The handedness of solenoid fiber is changed; CIDS and total scattered intensity for oppositely-handed chromatin helical fiber is given: (**a**,**b**) at a fixed orientation, (**c**,**d**) in the orientation average. λ = 300 nm.

Changing the handedness of chromatin solenoid, its effect on the angular behavior of differential scattering signal, and the unpolarized light scattering is demonstrated in Figure 5. Only the handedness is changed, and all other parameters are the same such as diameter, pitch, and one turn of chromatin fiber is modeled. It is observed the CIDS signal for oppositely handed helical fibers has the same angular behavior; however, opposite in sign with the same magnitude of scattering signal. In contrast, the total scattered intensity for the oppositely handed fibers has the same magnitude and same sign, as the scattered intensity is related to the overall size, and there is no effect on the scattered intensity for the changes in the handedness of solenoid. These observations are consistent at a fixed orientation of solenoid and in the orientation average (Figure 5).

**Figure 6:**
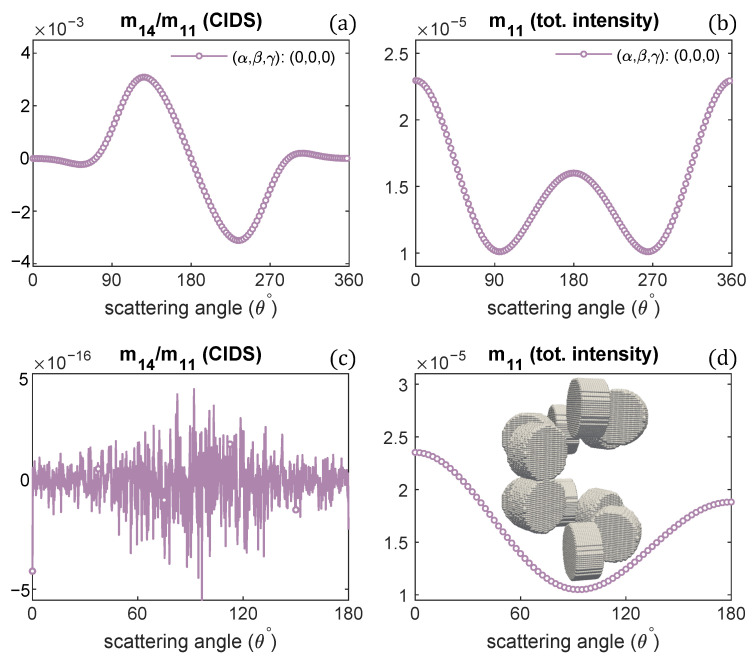
CIDS and total scattered intensity for the helical fiber as shown in the inset of (**d**): 1-turn of left-handed fiber on a 1-turn right-handed helical fiber; (**a**,**b**) at a fixed orientation, (**c**,**d**) in the fixed orientation.

The scattering calculations in Figure 5a,b advise the CIDS signal becomes opposite in sign as the handedness of fiber is changed. To get further insight and the manifestation of this observation, we modeled a chromatin helical fiber composed of one-turn with a right-handed solenoid and, on top of it, one-turn of a left-handed solenoid. The discrete dipole model of this system is shown in the inset of Figure 6d.

The two oppositely handed turns in this system have the same structural parameters; only the handedness is different. The scattering calculations are described in the Figure 6. The results indicate the CIDS signal is identically zero (Figure 6c) in the orientation average, as in this 2-turns chromatin fiber, one turn is a right-handed and the second turn is left-handed, and they have CIDS signal in the opposite direction that makes the overall CIDS signal zero. In comparison, the total scattered intensity is non-zero as it is affected by the changes in overall size and is not affected by the handedness (Figure 6d). However, in a fixed orientation, we can expect a non-zero CIDS signal as the calculations suggest (Figure 6a).

**Figure 7:**
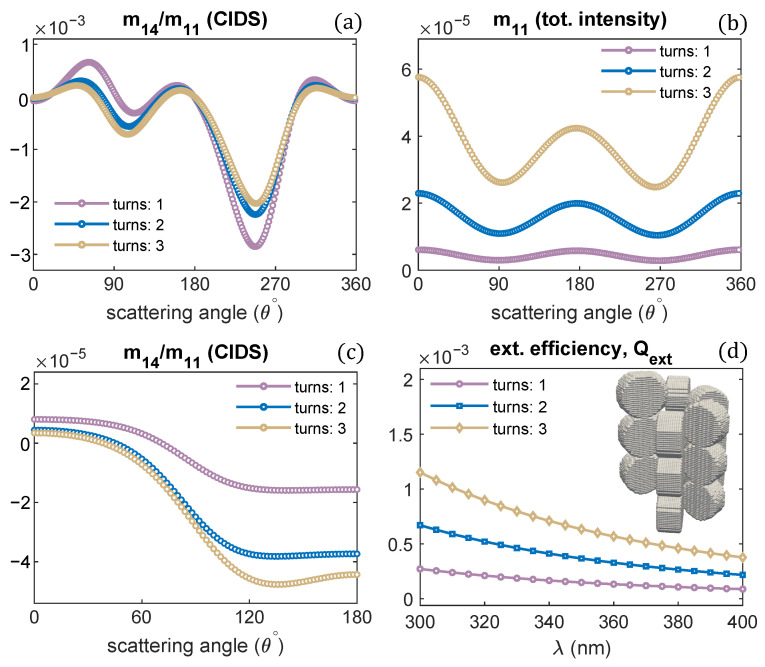
Solenoid helical turns are varied; (**a**,**b**) angular behavior of scattering quantities at fixed orientation (α,β,γ) = (0,0,0); and (**c**,**d**) in the orientation average. P = 11 nm, R = 10 nm, λ = 300 nm.

A single turn of the solenoid was examined in all the previous computations. The effect of varying the number of solenoid turns on the CIDS signal, and total scattered intensity is demonstrated in Figure 7a,b. Only the number of turns is varied, and all other structural parameters are the same as we used for one turn of a solenoid. The CIDS signal is largest in magnitude for a single turn of the solenoid at the simulated fixed orientation. In comparison, the total scattered intensity is smaller in magnitude for one turn of a solenoid, and it increases as the number of solenoid turns increases (Figure 7b). In the orientation averaging, we observe increasing the number of turns while keeping pitch and radius constant the magnitude of CIDS signal increases for this chromatin model (Figure 7c). The extinction efficiency of chromatin solenoid for the changes in its number of turns is given in Figure 7d; the calculations show that the extinction efficiency increases as the number of turns increases.

**Figure 8:**
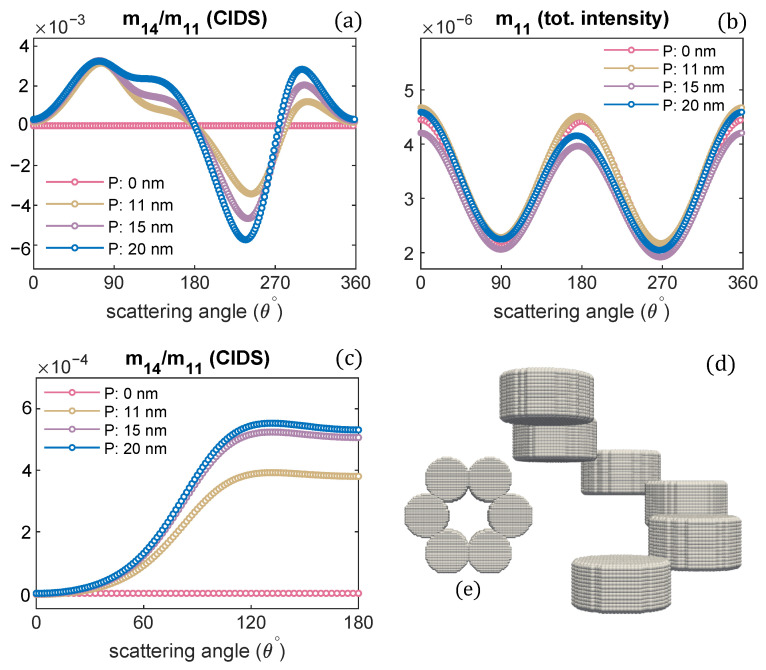
Varying pitch the scattering calculations are given: (**a**,**b**) at a fixed orientation (α,β,γ) = (0,0,0), (**c**) and in the orientation average, (**d**,**e**) chromatin fiber and its projected view looking along the helix axis.

Furthermore, changing the orientation of nucleosomes in reference to the helical axis, its effect on the circular differential scattering is demonstrated in Figure 8.

The chromatin model is shown in Figure 8d. A single turn of a solenoid is modeled. Only the orientation of nucleosomes is changed; all other structural parameters are kept the same as we used for the solenoid model in Figure 1. It is observed the CIDS senses the change in the orientation of nucleosomes as indicated by the angular behavior of CIDS signal in Figure 8a,c; the CIDS angular behavior is different from the CIDS signal of chromatin fiber with the nucleosomes orientation as modeled in Figure 2a,c. However, the angular behavior of total scattered intensity is similar for both chromatin models. The calculations in Figure 8a,c also indicate the magnitude of CIDS is significant when the pitch is largest, and it is identically zero when the pitch is zero; this behavior is in agreement with the differential scattering signal in Figure 2a,c.

**Figure 9:**
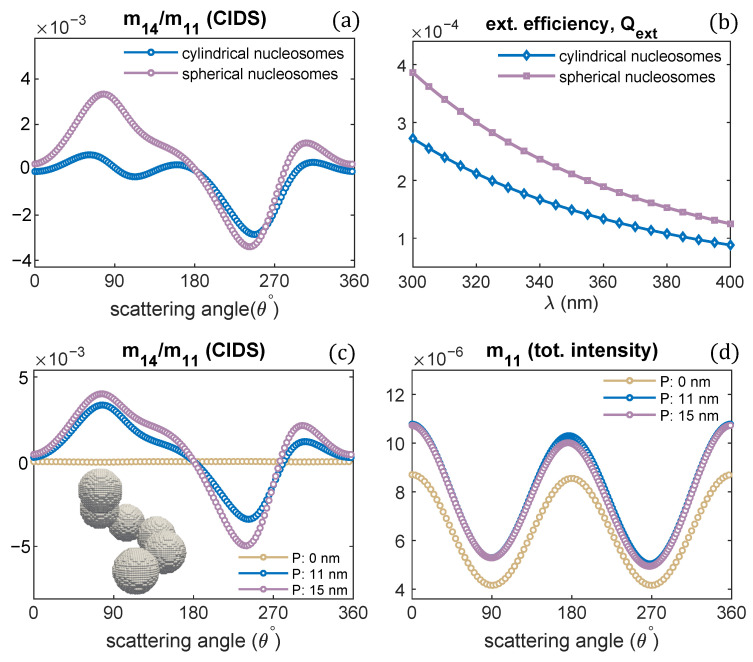
(**a**,**b**) CIDS and extinction efficiency for two different shaped nucleosomes, respectively; (**c**,**d**) scattering calculations for the chromatin fiber with spherical shaped nucleosomes, λ = 300 nm.

The effect of nucleosomes shape on the CIDS signal has been described in Figure 9. The nucleosomes have been modeled as cylindrical units and spheres. The chromatin solenoid with cylindrical shape nucleosomes is the same as we modeled in Figure 1. The spherical nucleosomes have a diameter of 11 nm. The 30 nm chromatin solenoid with pitch 11 nm is considered for these calculations. The calculations show that solenoid with spherical nucleosomes has a strong CIDS signal than the solenoid with cylindrical. The solenoid with spherical units also has higher extinction efficiency than the extinction efficiency of the solenoid with cylindrical nucleosomes (Figure 9b). Within the first Born approximation, the zero CIDS signal is expected for the solenoid composed of spheres; as for chiral scatterers, this approximation requires anisotropic polarizability tensor and gives zero CIDS for isotropic scatterers [29]. A significant CIDS signal exists for the chromatin fiber composed of spherical nucleosomes as computed by the discrete dipole approximation (Figure 9).

## 4. Conclusions

In this paper, the angular behavior of the scattering quantities were demonstrated in response to the changes in various chiral structural parameters of the chromatin solenoid; these changes in parameters were related to different compaction levels of chromatin. The differential scattering of circularly polarized light (CIDS) sensed the changes in the chiral parameters and exhibited particular angular behavior representing the fingerprint of a chromatin model. For the oppositely handed helical fibers the CIDS signal showed same angular behavior, however, with the opposite sign; this observation triggered to model a solenoid system composed of both left-handed and right-handed helical fibers and found the CIDS signal was identically zero in the orientation average; this implies CIDS signal can be used to find the average handedness of the helical fiber. The CIDS sensitivity to the shape of the nucleosomes, cylindrical and spherical was also demonstrated. The total scattered intensity calculations gave information about the overall size of the solenoid structures. However, the CIDS provided information differently from the total scattered intensity and described the chirality of the solenoids. The scattering calculations were performed using the discrete dipole approximation method. These calculations can potentially benefit in a label-free characterization of chiral polymers in biophysics and nano-chemistry.

## Data Availability

The data presented in this study are available on request from the corresponding author.

## Abbreviations

The following abbreviations are used in this manuscript:

CIDS	Circular intensity differential scattering
CD	Circular dichroism
DDA	Discrete dipole approximation
ADDA	DDA code
LCP	Left circularly polarized
RCP	Right circularly polarized
